# Mass transfer kinetics (soluble solids gain and water loss) of ultrasound-assisted osmotic dehydration of apple slices

**DOI:** 10.1038/s41598-022-19826-w

**Published:** 2022-09-13

**Authors:** Fakhreddin Salehi, Rana Cheraghi, Majid Rasouli

**Affiliations:** 1 Department of Food Science and Technology, Bu-Ali Sina University, Hamedan, Iran; 2 Faculty of Agriculture, Bu-Ali Sina University, Hamedan, Iran

**Keywords:** Chemical engineering, Chemical physics

## Abstract

Ultrasound (sonication) treatment can be used directly for dehydration or pre-treatment before the osmotic dehydration (OD) procedure of fruit or vegetable particles. The combination of this technique with the OD technique can further improve the dehydration process efficiencies by increasing the mass transfer rates and enhancing final product quality. In this study, apple slices were osmotically dehydrated in different hypertonic sucrose solutions and assisted with or without ultrasound. Sucrose concentrations (in three levels of 30, 40, and 50° Brix), sonication power (in three levels of 0, 75, and 150 W), and treatment time (in six time intervals: 10, 20, 30, 40, 50, and 60 min) were the factors investigated concerning weight reduction, soluble solids gain, water loss and rehydration. Also, mass transfer kinetics were modelled according to Page, Newton, Midilli, Logarithmic, Verma, and Two terms equations. Increased sucrose solution concentration resulted in higher weight reduction, soluble solids gain and water loss. Also, increased sonication power levels resulted in higher weight reduction, soluble solids gain and water loss. The average rehydration ratio of apple slices decreased from 237.7 to 177.5%, by increasing osmotic solution concentration from 30 to 50%. The Page equation showed the best fitting for water loss data. The effective moisture diffusivity (D_eff_) of apple slices during OD calculated using Fick’s second law applied to a slab geometry was found to be in the range of 1.48 × 10^–10^ and 4.62 × 10^–10^ m^2^s^−1^ for water loss.

## Introduction

OD is an easy method for removing water from fruit and vegetable particles. At the same time, the correct term is “osmotic dewatering” while the final product still has high water content. This partial dewatering is done by plunging the fruit or vegetable particles in a concentrated aqueous solution. This process has been found to be effective even at ambient temperature and it is generally used as a pre-treatment before drying process. The relation of OD is mainly related to enhancing some physicochemical, nutritional, functional, and sensorial characteristics of the dehydrated product^1–3^. The combination of ultrasound technique with the OD process can further improve the dehydration process efficiencies by increasing the mass transfer rates and enhancing final product quality^4^.

The term ultrasound (sonication) demonstrates acoustic longitudinal waves with a frequency above the threshold of human hearing (20 kHz). The ultrasound technique is cost-effective, simple, non-invasive, energy-saving and is emerging tremendously^5^. Also, this technique is a powerful synthesis method for a multitude of nanobiomaterials^6^. Ultrasound treatments support the removal of intracellular water from fruit or vegetable particles to the surroundings as a result of a quick series mechanism of compressions and expansions (the phenomenon of cavitation)^3,7^. This technique has the combined impacts of osmotic pressure gradient and sonication effects on the surface of the fruit and vegetable pieces which disarranged the cells structure by producing micro channels within cells structure and resulting in higher moisture loss^8,9^. Also, reduction of dehydration time and, as a result, processing costs have lately been reported at the laboratory scale after research conducted on some fruit and vegetable particles^10–12^.

The various ultrasound-assisted OD conducted in multiple fruit and vegetable particles such as banana^4^, kiwifruit^13–15^, persimmon^16^, apples^1,3^, papayas^17^, strawberry^12^, plum^18^ and cranberry^19^ were increased the water loss rates and reduce the dehydration time. Nowacka et al.^11^ examined the use of sonication as a mass transport improving technique before dehydration of apple slices. The application of ultrasound caused a decrease of the drying time of about 31–40% compared to untreated samples. The impact of ultrasound application on the water state in kiwifruit slices during OD was investigated by Nowacka et al.^13^. The results confirmed that ultrasound application performed for more than 10 min has a positive impact on the mass exchange caused by OD. In another study, the impact of sonication (35 kHz for 10, 20, and 30 min) and OD treatment (30 °C and 45° Brix) on convective drying and quality characteristics of persimmon fruit was studied by Bozkir et al.^16^. This procedure increased water reduction and soluble solids gain of persimmon samples. In another study, Tao et al.^20^ investigated on the performances of air drying of blackberries assisted by airborne ultrasound and contact ultrasound. The results showed that the blackberries dehydrated by contact ultrasound contained more anthocyanins and organic acids than those dried by airborne ultrasound, implying a higher quality. It should, however, be noted that for our current knowledge, no paper has comprehensively examined and modelled the applications of ultrasound-assisted OD treatment, as well their potential to improve the OD process efficiencies of apple slices. Therefore, the objective of this study was to examine the influence of the use of ultrasound during OD in sucrose solution on the mass transfer (solid gain and water loss) from apple slices. Also, the impacts of ultrasound treatments on weight reduction, rehydration percentage and D_eff_ during the process were also investigated.

## Materials and methods

### Samples preparation

The collection of apples in this study was done according to legislation and formal permission of Iran National Standards Organization. Fresh apples of the *Golden delicious* variety were harvested in a patch located in Maragheh, East Azerbaijan Province, Iran. Before the experiments, the fresh apples were washed, and with the aid of a sharp knife and a cylindrical shape mold cut into 0.5 cm thickness slices. The fresh apple slices moisture content (MC) was 85.7% w.b. (was calculated at 105 °C for four h, in an air forced oven, Shimaz, Iran).

### Ultrasound-assisted OD procedure

The ultrasonic treatments were carried out using an ultrasonic bath (vCLEAN1-L6, Backer, Iran; internal dimensions: 33 × 18 × 31 cm; volume: 6L). The operating frequency of the bath was 40 kHz.

Treatments were structured in combinations of six time intervals: 10, 20, 30, 40, 50, and 60 min; three osmotic solutions concentrations: 30, 40, and 50° Brix (at 50 °C); and three sonication power levels: 0, 75, and 150 watts. Treatments performed at 0 W were not subjected to sonication and were considered as control samples.

The osmotic solutions were prepared with the addition of food-grade sucrose to water until concentrations of 30, 40, and 50° Brix (% w/w) were attained. Each apple slice was immersed in the ultrasonic bath filled with 3L of treatment solution. Weights for each sample before and after ultrasound-assisted OD treatment were recorded. Then, to calculate the amount of moisture loss and solids gain, pieces removed from the osmotic solutions were placed in an oven at 105 °C until reaching a constant weight. All experiments were carried out in 3 replicates.

### Calculation of process efficiency parameters

The response variables of weight reduction (WR), soluble solids gain (SG), and water loss (WL) were calculated using the weight of apple slices before and after the treatment experiments, as well as the moisture content (w.b.) and total solids of apple slices before and after treatment (was calculated at 105 °C for 4 h, in an air forced oven, Shimaz, Iran). WR, SG and WL were determined according to Eqs. 1, 2 and 3, respectively^4^.

1 $$ WR = \frac{{A_{0} - A_{t} }}{{A_{0} }} \times 100 $$

2 $$ SG = \frac{{S_{t} - S_{0} }}{{A_{0} }} \times 100 $$

3 $$ WL = \frac{{W_{0} - W_{t} }}{{A_{0} }} \times 100 = \frac{{W_{0} - (A_{t} - S_{t} )}}{{A_{0} }} $$

where A_0_ is the fresh apple slice weight (g) before treatments; A_t_ is the final apple slice weight (g) after treatments; W_0_ is the fresh apple slice moisture content before treatments (g; w.b.); W_t_ is the final apple slice moisture content after treatments (g; w.b.); S_0_ is the fresh apple slice dry solid matter content (g) before treatments; and S_t_ is the final apple slice dry matter content (g) after treatments.

### Rehydration of dried apple slices

After each treatment, the apple slices were placed in aluminium foil in a single layer arrangement and dried in a forced circulation air drying oven (Shimaz, Iran). Rehydration trials were performed carried out at 50 °C for 20 min. These trials were carried out with a water bath. 200 mL water was added into a 250 mL glass container. Based on water absorption during the rehydration procedure, the mass of the dried pieces increases^21^. So, the rehydration ratio (RR) of apple pieces was estimated by using Eq. (4).

4 $$ RR = \frac{{M_{r} }}{{M_{0} }} \times 100 $$

M_r_ and M_0_ are the weight of the rehydrated apple slice and the weight of the dry apple slice (after oven), respectively.

### Mathematical modeling of dehydration kinetics

For numerical modelling of the dehydration kinetic behavior of apple slices (during OD with or without ultrasound), six typically thin layer equations include Page, Newton, Midilli, Logarithmic, Verma, and Two terms were used^22^. In these equations, dimensionless moisture ratio (MR) were defined as Eq. (5):

5 $$ MR = \frac{{M_{t} - M_{e} }}{{M_{0} - M_{e} }} $$

where MR is the dimensionless moisture ratio, M_t_, M_0_, and M_e_ are moisture content of apple slices at a given time, initial moisture content and equilibrium moisture content of apple slices (g water/g dry matter), respectively. In Eq. (5), since M_e_ <  < M_t_ and M_e_ <  < M_0_, the value of M_e_ is insignificant and the equation was abbreviated to M_t_/M_0_.

Matlab software (version R2012a) was used to estimate the coefficients of these equations. The three criteria used to evaluate the adjustment of the experimental data were the square of the correlation between the response data and the predicted response data (R-square or R^2^), and the sum of squares due to error (SSE) and root mean squared error (RMSE).

### Calculation of effective moisture diffusivity (D_eff_)

According to Fick’s second law, moisture ratio for various geometries, including cylinder, slab, and sphere is described as Eq. (6):

6 $$ \frac{\partial MR}{{\partial t}} = D_{eff} \nabla^{2} MR $$

D_eff_ was based on the simplified Fick’s second law for slab geometry equation (Eq. 7):

7 $$ MR = \frac{8}{{\pi^{2} }}\sum\nolimits_{n = 1}^{\infty } {\frac{1}{{(2n + 1)^{2} }}} \exp \left( { - (2n + 1)^{2} \frac{{\pi^{2} D_{eff} t}}{{4L^{2} }}} \right) $$

where t is the dehydration time (s), D_eff_ is the effective moisture diffusivity (m^2^s^−1^); L is half-thickness of apple slice, which is equal to 0.25 × 10^–2^ m in this research.

For long dehydration procedure duration, Eq. (7) can be further simplified to:

8 $$ MR = \frac{8}{{\pi^{2} }}\exp \left[ {\frac{{ - \pi^{2} D_{eff} t}}{{4L^{2} }}} \right] $$

For this reason, a logarithmic type was presented as follow:

9 $$ LnMR = Ln(\frac{8}{{\pi^{2} }}) - \frac{{\pi^{2} D_{eff} t}}{{4L^{2} }} $$

The D_eff_ was estimated through equation nine by using the technique of slopes. From Eq. (9), a plot of empirical dehydration data in term of lnMR versus dehydration duration give a straight line with a slope (K) of^23^:

10 $$ Slope(K) = - \frac{{\pi^{2} D_{eff} }}{{4L^{2} }} $$

### Ethical approval and consent to participate

This study does not involve any human or animal testing.

## Results and discussion

### Weight reduction (WR)

**Figure 1:**
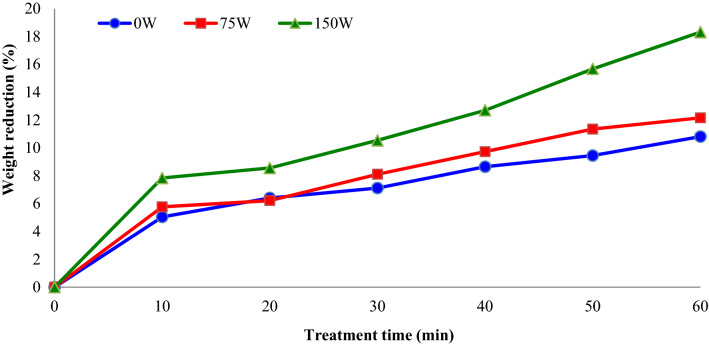
Impacts of sonication power and treatment time on the weight reduction (%) of apple slices during osmotic dehydration (concentration = 40° Brix).

Sonication procedure can be used directly for dehydration or as a pre-treatment before the OD process. Figure 1 shows variations of weight reduction (%) of apple slices during OD. As seen from this figure, sonication power play an essential role in weight reduction. It was considered that weight reduction increased with the enhancement in sonication powers. When ultrasound power increased to 75 and 150 W, reductions in apple slices weight (after 60 min) were 12.5 and 50.6%, respectively. The highest ultrasound power, the highest weight reduction and the slowest drying duration. Weight reduction was caused by the influence of sonication, which simplified the removal of water from the apple slices. Higher weight reduction of apple slices during plunging with ultrasound using confirmed that sonication waves influenced water removal from the interior of apple slices. Overall these findings are in accordance with findings reported by Kroehnke et al.^15^, where treatment with ultrasound (25 kHz) increases mass transfer during OD and reduces the drying time of kiwifruit.

**Figure 2:**
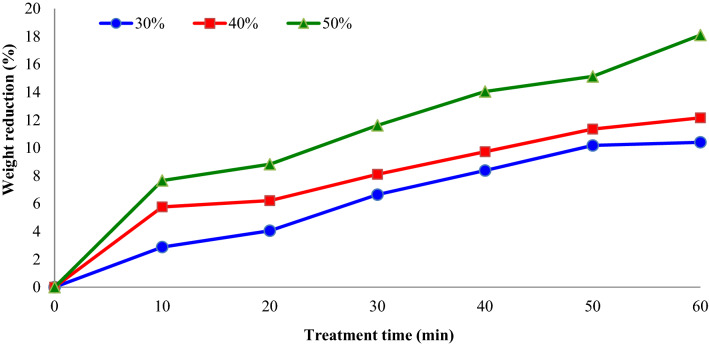
Impacts of sucrose solution concentration (°Brix) and treatment time on the weight reduction (%) of apple slices during osmotic dehydration (sonication power = 75 W).

Figure 2 shows the impacts of osmotic solution concentration (°Brix) and treatments time on the weight reduction (%) of apple slices during OD. It was observed that weight reduction increased with the enhancement in osmotic solution concentration from 30 to 50%. Furthermore, during a long time of plunging in sucrose solution, water penetrates into the apple slices due to osmotic solution concentration differences. Fijalkowska et al.^3^ studied the effect of sonication pre-treatment (21 and 35 kHz for 30 min) on drying kinetics and physicochemical characteristic of dried apple pieces. They reported that the ultrasound pre-treatments declined the drying duration of apple slices by 13–17% in comparison with the untreated slices.

### Soluble solids gain (SG)

**Figure 3:**
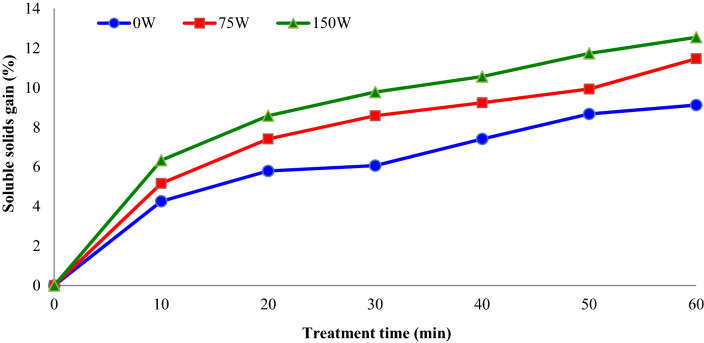
Impacts of sonication power and treatment time on the soluble solids gain (%) of apple slices during osmotic dehydration (concentration = 40° Brix).

The impacts of sonication power and treatment time on the soluble solids gain of ultrasound-assisted osmotically dehydrated apple slices are demonstrated in Fig. 3. As understood from this figure, soluble solids gain percentage of ultrasound-assisted osmotic dehydrated apple slices (75 and 150 W) was found to be higher than osmotic dehydrated apple slices (0 W). In the ultrasonic-assisted OD (sonication power = 150 W) the apple slices gained 12.5% of sucrose after 60 min and in the OD without sonication treatment, the apple slices gained 9.1% of sucrose in the 60 min.

**Figure 4:**
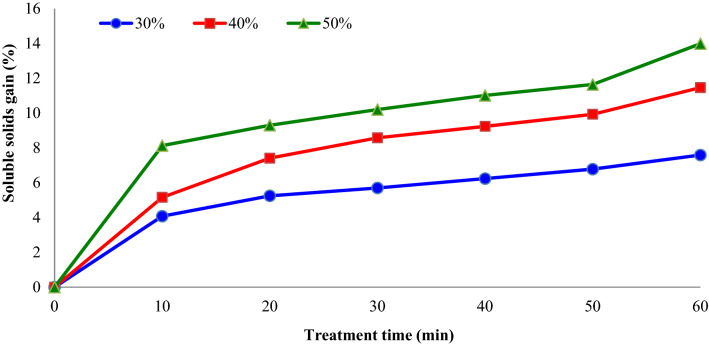
Impacts of sucrose solution concentration (°Brix) and treatment time on the soluble solids gain (%) of apple slices during osmotic dehydration (sonication power = 75 W).

Figure 4 demonstrate the impacts of osmotic solution concentration (°Brix) and treatment time on the soluble solids gain (%) of dehydrated apple slices. The average soluble solids gain (%) of apple slices increased from 5.1 to 9.2% with increasing osmotic solution concentration from 30 to 50%. As well, with increasing treatments time, the soluble solids gain (%) of apple slices increased. The results confirmed that the samples submitted to OD treatment (with or without ultrasound) for 10 min and for 60 min have minimum and maximum solids absorption, respectively.

### Water loss (WL)

**Figure 5:**
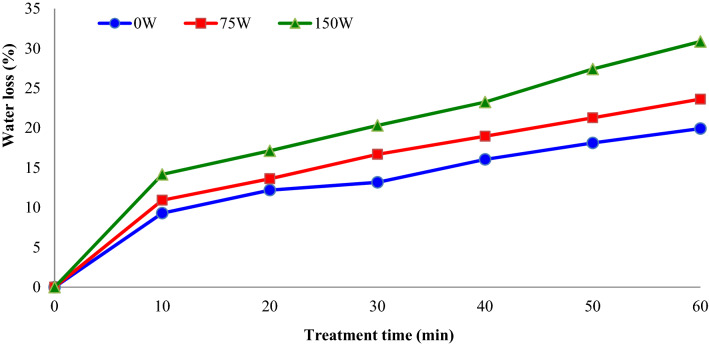
Impacts of sonication power and treatment time on the water loss (%) of apple slices during osmotic dehydration (concentration = 40° Brix).

Ultrasound in mixture with high sucrose concentration accelerate the velocity of water withdrawal from the apple slices and reduces the dehydration duration. Figure 5 shows the effect of sonication power and treatment time on the variations of water loss (%) of apple slices during OD. As seen from this figure, sonication power play a significant function in water loss. It was confirmed that water loss increased with the enhancement in sonication powers. When ultrasound power increased to 75 and 150 W, reductions in apple slices moisture content (after 60 min) were 18.5 and 30.6%, respectively. The higher the ultrasound power, the higher the water loss and the lower the dehydration time.

**Figure 6:**
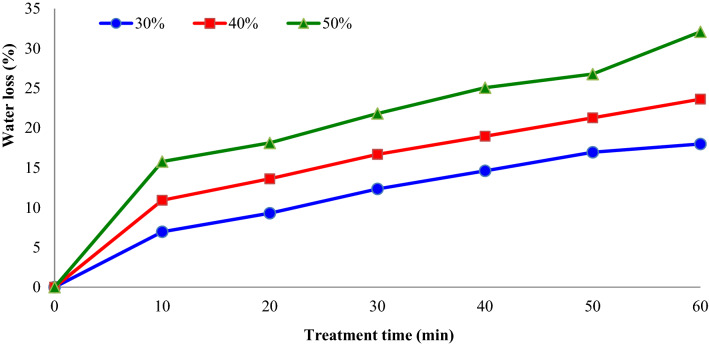
Impacts of sucrose solution concentration (°Brix) and treatment time on the water loss (%) of apple slices during osmotic dehydration (sonication power = 75 W).

The water loss during the OD procedure is mainly influenced by osmotic solution types and their concentration^18^. Figure 6 shows the impacts of osmotic solution concentration (°Brix) and treatment time on the water loss (%) of apple slices during OD. It was observed that water loss increased with the enhancement in osmotic solution concentration from 30 to 50%. The time required to decrease the water content of apple slices about by 18% (w.b.) was found as 60, 35 and 20 min for 30° Brix, 40° Brix and 50° Brix, respectively. In another study, Garcia-Noguera et al.^12^ investigated the impacts of pre-treatment time (10, 20, 30, and 45 min) and sonication frequency (0, 25, and 40 kHz) and osmotic solution concentration (0, 25, and 50% (w = w)) on water loss and soluble solids gain of strawberry halves. The authors reported that higher amounts of water were lost from a treatment occurring for samples at 25 kHz between 10 and 20 min than for other times.

### Rehydration ratio

**Figure 7:**
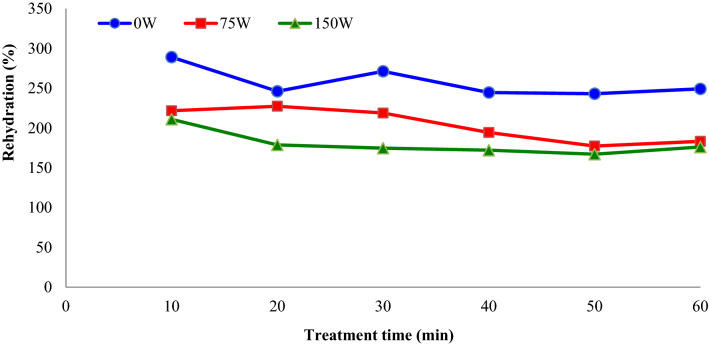
Impacts of sonication power and treatment time on the rehydration (%) of dried apple slices (concentration = 40° Brix).

**Figure 8:**
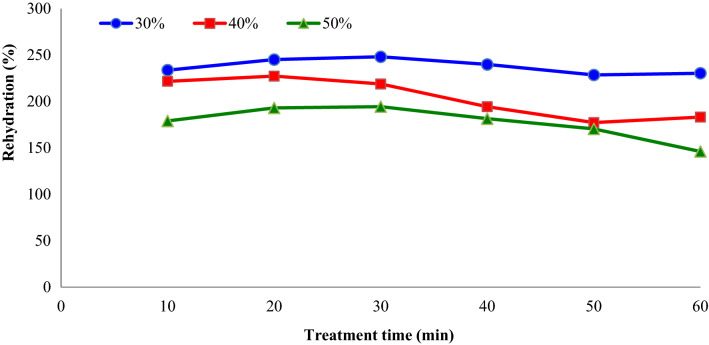
Impacts of sucrose solution concentration (°Brix) and treatment time on the rehydration (%) of dried apple slices (sonication power = 75 W).

The rehydration procedure is mainly carried out before utilizing dehydrated fruit and vegetable products. Rehydration is the procedure of regaining water to dehydrated products ^21^. The effect of sonication power and treatment time on the rehydration ratio of ultrasound-assisted osmotic dehydrated apple slices are shown in Fig. 7. As understood from this figure, rehydration ratio of ultrasound-assisted osmotic dehydrated apple slices (75 and 150 W) was found to be lower than osmotic dehydrated apple slices (0 W). This can be explain with the contraction of the samples, which is caused by ultrasound. In addition, Fig. 8 shows the effect of osmotic solution concentration (°Brix) and treatment time on the rehydration (%) of dried apple slices. The average rehydration ratio of apple slices decreased from 237.7 to 177.5%, by increasing osmotic solution concentration from 30 to 50%. The use of ultrasound and curing agents during OD for enhancing the quality characteristics of freeze-dried yellow peach slices was examined by Chu et al.^24^. The authors reported that with increasing ultrasound treatment time, the drying time and shrinkage of the product would reduce. Also, compared with direct dried samples, the rehydration capacity of the pre-treated samples was increased, the colour and nutritional elements can be preserved better and the textural properties were further enhanced.

### Dehydration kinetics

**Figure 9:**
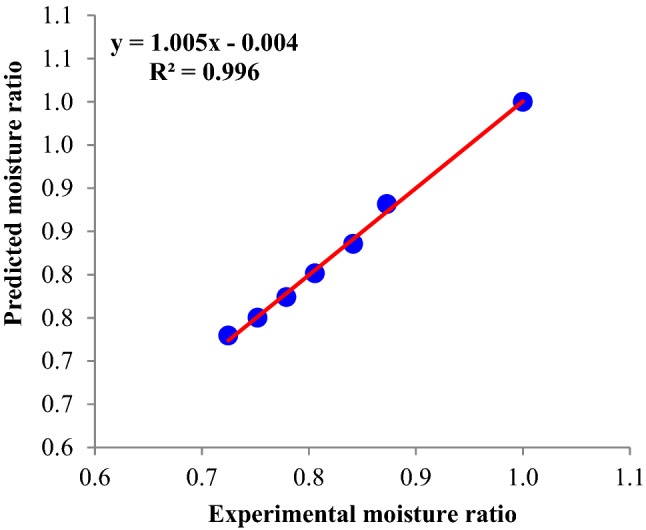
Evaluation of fitted data by Page model with empirical results (sonication power = 75 W and osmotic solution concentration = 40° Brix).

The drying process of various fruit and vegetable products can be modeled to estimate the drying process parameters of the samples based on the pattern achieved from the equation and also to choose the appropriate operating conditions for dehydration of fruit and vegetable samples^25^. The kinetics model with the highest R^2^ value and the lowest SSE and RMSE values was selected as an appropriate model to modelling the OD procedure of apple slices. The model that satisfied these features was the Page model (Eq. 11):

11 $$ {\text{MR}} = \exp \left( { - {\text{kt}}^{{\text{n}}} } \right) $$

where MR and t are moisture ratio and dehydration time of apple slices at various sonication power levels and osmotic solution concentrations, respectively. The calculated parameters (fitting data) of the Page model, including dehydration constants, k, and n, are reported in Table 1 along with corresponding statistical data (R^2^, SSE and RMSE) for all experiments conditions. The R^2^ values for all experiments were in the ranges of 0.972–0.996. Also, the values of SSE and RMSE for all conditions were in the ranges of 0.0001–0.0043 and 0.0048–0.0292, respectively. Figure 9 demonstrates the evaluation of fitted moisture ratio data by Page model with empirical data (sonication power = 75 W and osmotic solution concentration = 40° Brix). These results indicate that the Page equation is a suitable to model in describing the OD procedure of apple slices under the various sonication power levels and osmotic solution concentrations. The influence of ultrasonic pre-treatment on drying kinetics and physio-mechanical properties of peach slices was examined and modeled by Akhoundzadeh Yamchi et al.^26^. According to the results, applying sonication before drying decreases the drying duration about 40%. Also, the midilli equation in various pre-treatments, as compared to other equations, had the greatest fitting with the experiential data.

**Table 1:** Parameters of Page model describing the kinetics of osmotic dehydration rate of apples slices.

Sonication power (W)	Concentration (°Brix)	k	n	R^2^	SSE*	RMSE**
0	30	0.00456	0.9381	0.986	0.0004	0.0087
75	30	0.0186	0.6246	0.996	0.0001	0.0048
150	30	0.0379	0.446	0.972	0.0008	0.0131
0	40	0.0351	0.4837	0.989	0.0004	0.0086
75	40	0.0387	0.5124	0.993	0.0003	0.0084
150	40	0.0428	0.5597	0.989	0.0009	0.0132
0	50	0.0352	0.6052	0.979	0.0017	0.0185
75	50	0.0564	0.4944	0.985	0.0013	0.0159
150	50	0.0367	0.7112	0.976	0.0043	0.0292

*The sum of squares due to error (SSE).

**Root mean squared error (RMSE).

### Effective moisture diffusivity (D_eff_)

**Figure 10:**
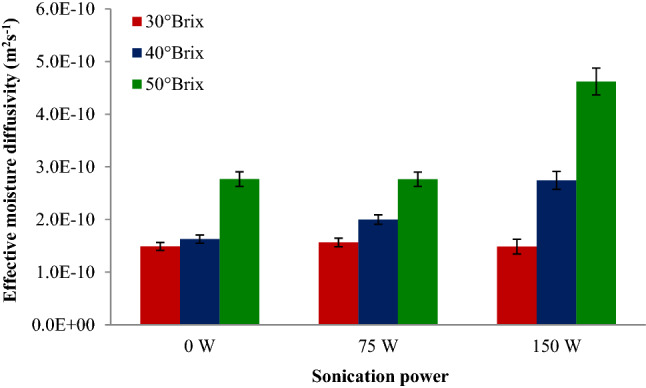
The interaction impacts of sonication power levels and osmotic solution concentrations on the effective moisture diffusivity (D_eff_) values of apple slices during osmotic dehydration.

The D_eff_ values during dehydration and drying procedures of fruits and vegetables lie within the range of 10^–8^–10^–10^ m^2^s^−1^^27^. The D_eff_ values are calculated by drawing empirical dehydration data regarding lnMR versus time. The impact of sonication power levels and osmotic solution concentrations on the D_eff_ values of apple slices during OD are shown in Fig. 10. In this study, the D_eff_ values of apple slices during the dehydration process were ranged from 1.48 × 10^–10^ and 4.62 × 10^–10^ m^2^s^−1^. D_eff_ values increased with the increment of sonication power levels because of the rapid water extract at high sonication power. The average D_eff_ values increased from 1.96 × 10^–10^ to 2.95 × 10^–10^ m^2^s^−1^ with increasing sonication power level from 0 to 150 W. In addition, the average D_eff_ values increased with increasing osmotic solution concentration and they were equal to 1.51 × 10^–10^, 2.12 × 10^–10^ and 3.38 × 10^–10^ m^2^s^−1^ for 30, 40 and 50° Brix, respectively. The use of ultrasound treatment to increase mass transfer rates during OD of apple cubes was examined by Simal et al.^28^. The authors report the D_eff_ values for apple cubes in 70° Brix sucrose solution, in the range from 2.6 × 10^–10^ m^2^s^−1^ at 40 °C to 6.8 × 10^–10^ m^2^s^−1^ at 70 °C.

## Conclusion

In this study, we examined the impacts of the osmotic procedure with or without ultrasound on the mass transfer kinetics (weight reduction, soluble solids gain, water loss and rehydration) and D_eff_ of apple slices. It was observed that weight reduction increased with the enhancement in osmotic solution concentration from 30 to 50%. When ultrasound power increased to 75 and 150 W, reductions in apple slices weight (after 60 min) were 12.5 and 50.6%, respectively. The average soluble solids gain (%) of apple slices increased from 5.1 to 9.2% with increasing osmotic solution concentration from 30 to 50%. When ultrasound power increased to 75 and 150 W, reductions in apple slices moisture content (after 60 min) were 18.5 and 30.6%, respectively. The average rehydration ratio of apple slices decreased from 237.7 to 177.5%, by increasing osmotic solution concentration from 30 to 50%. The water loss kinetics were acceptably modeled by the Page equation with the highest R^2^ values (higher than 0.972) and the lowest SSE values (lower than 0.004) and RMSE values (lower than 0.029). Values for the D_eff_ of apple slice samples during OD were obtained in the range of 1.48 × 10^–10^ and 4.62 × 10^–10^ m^2^s^−1^; and the D_eff_ values were increased with increasing sonication power levels from 0 to 150 W and osmotic solution concentrations from 30 to 50° Brix.

## Data Availability

All data generated or analysed during this study are included in this published article. All authors read and approved the final manuscript.

## Abbreviations

D_eff_: Effective moisture diffusivity
MC: Moisture content
OD: Osmotic dehydration
RMSE: Root mean squared error
SSE: Squares due to error
